# Reasons for new MIS. Let's be fair: iTIND, Urolift and Rezūm

**DOI:** 10.1007/s00345-020-03453-z

**Published:** 2020-09-22

**Authors:** Rodrigo Suarez-Ibarrola, Arkadiusz Miernik, Christian Gratzke, Dominik S. Schoeb

**Affiliations:** Department of Urology, Faculty of Medicine, University of Freiburg–Medical Centre, Freiburg, Germany

**Keywords:** (MeSH terms of the US national library of medicine), Male urologic surgical procedures, Bladder outlet obstruction, Benign prostatic hyperplasia, Urinary tract disease, Minimal invasive surgical procedures

## Abstract

**Purpose:**

To review and discuss the literature regarding iTIND, Urolift and Rezūm and investigate the precise clinical indications of all three different approaches for their application in benign prostatic hyperplasia (BPH) treatment.

**Materials and methods:**

The PubMed–Medline and Cochrane Library databases were screened to identify recent English literature relevant to iTIND, Urolift and Rezūm therapies. The surgical technique and clinical results for each approach were summarized narratively.

**Results:**

iTIND, Urolift and Rezūm are safe and effective minimally invasive procedures for the symptomatic relief of lower urinary tract symptoms (LUTS) due to BPH. iTIND requires the results of ongoing prospective studies, a long-term follow-up and a comparison against a reference technique to confirm the generalizability of the first pivotal study. Urolift provides symptomatic relief but the improvements are inferior to TURP at 24 months and long-term retreatments have not been evaluated. Rezūm requires randomized controlled trials against a reference technique to confirm the first promising clinical results. However, clinical evidence from prospective clinical trials demonstrates the efficacy and safety of these procedures in patients with small- and medium-sized prostates.

**Conclusions:**

Although iTIND, Urolift, and Rezūm cannot be applied to all bladder outlet obstruction (BOO) cases resulting from BPH, they provide a safe alternative for carefully selected patients who desire symptom relief and preservation of erectile and ejaculatory function without the potential morbidity of more invasive procedures.

## Introduction

Benign prostatic hyperplasia (BPH) is a common ailment in urologic practice affecting up to 30% of men over 50 years [1, 2]. BPH causes physical compression of the urethra and results in bladder outlet obstruction (BOO) either through an increase in prostate volume or an increase in smooth muscle tone and is clinically characterized by lower urinary tract symptoms (LUTS) [3]. LUTS are known to substantially diminish patient’s health-related quality of life and are of significant socio-economic importance to public health systems worldwide considering the changing demographic landscape [4, 5].

Existing therapeutic strategies range from observation, medical treatment to a variety of surgical treatment modalities. Surgical intervention is appropriate in patients who failed medical treatment, present with moderate-to-severe LUTS, and have developed BPH-related complications such as urinary retention, bladder stones, recurrent urinary tract infections, and renal failure. Traditionally, transurethral resection of the prostate (TURP) has been the treatment method of choice and is still recommended in most national and international guidelines as the gold-standard for gland sizes of up to 80 cc. However, TURP is accompanied by a substantial perioperative morbidity rate of up to 20% [6] and postoperative complications include anejaculation (65%), erectile dysfunction (10%), urethral strictures (7%) and incontinence (3%) [7]. While the development of transurethral enucleation techniques using different energy sources such as holmium and thulium lasers have led to the replacement of simple prostatectomy and have become the standard for larger gland sizes, wherever the techniques are available, TURP is still applied to small and medium-size prostatic adenomas in most urological centers. Therefore, newer minimally invasive procedures (MIS) strive to rival standard BPH interventions by providing durable outcome efficacy and improved safety profiles.

In this study, we describe three promising minimally invasive treatment modalities (iTIND, Urolift and Rezūm) and review the current literature regarding their safety, functional outcome efficacy, and indications to be implemented in BPH treatment.

## Materials and methods

**Fig. 1:**
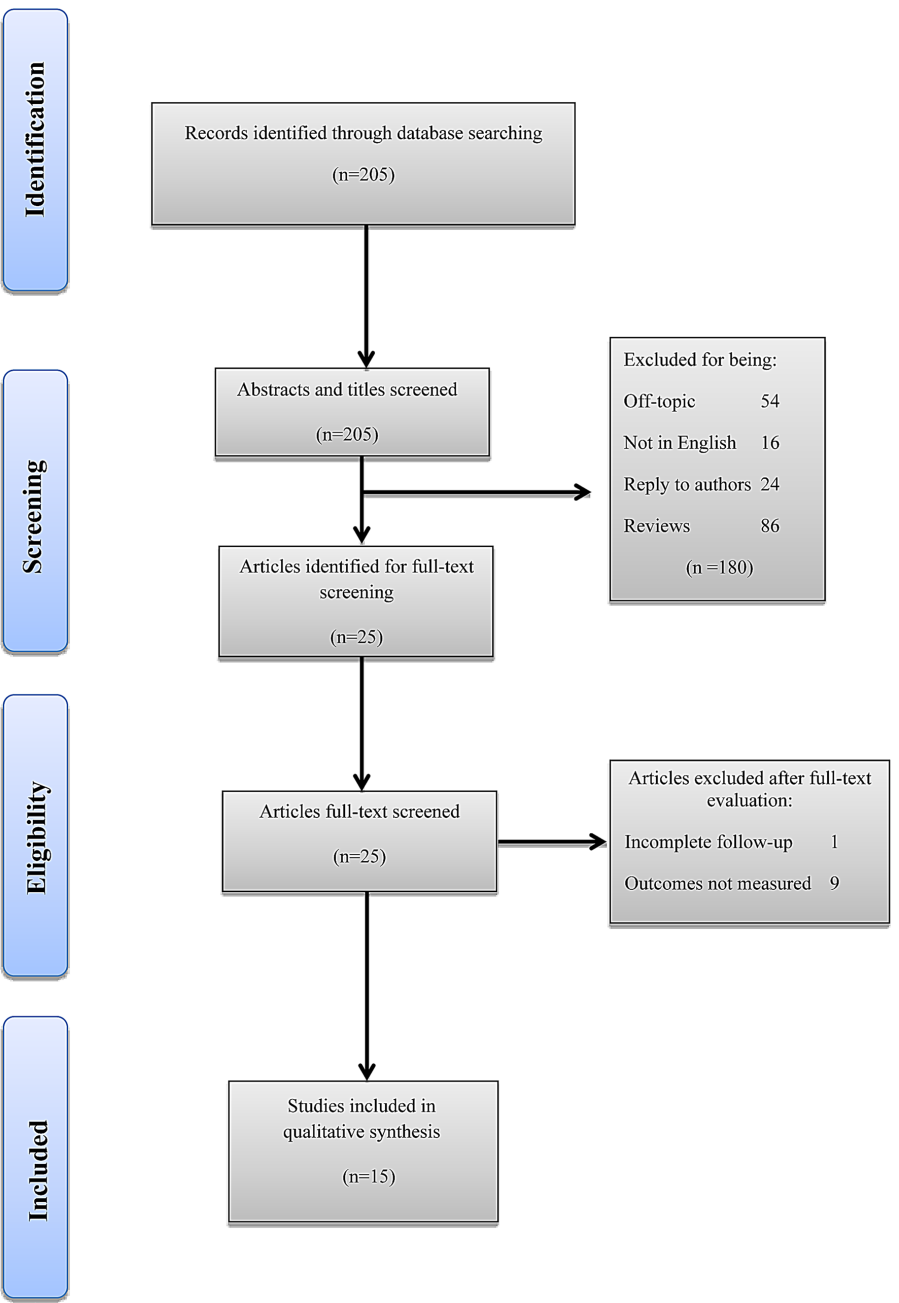
Preferred reporting items for systematic reviews and meta-analyses (PRISMA) flow diagram detailing the search strategy and identification of studies used in evidence synthesis

A non-systematic search was performed using the PubMed–Medline and Cochrane Library databases up to 4 August 2020 using the term “benign prostatic hyperplasia”, in combination with the following terms: “iTIND”, “temporary implantable nitinol device”, “Urolift”, “prostatic urethral lift”, “Rezum”, and “water vapor thermal therapy”. As proposed by the PRISMA guidelines, we used the Population, Intervention, Comparator, Outcomes and Study design approach to specify the eligibility criteria. Therefore, studies were considered eligible if BPH patients (population) were treated with iTIND, Urolift, or Rezūm (intervention), and compared to patients treated with TURP (comparator) or a single-arm study group to investigate urinary clinical outcomes. After article selection and according to the eligibility criteria, the following types of studies were excluded: articles not written in English, commentaries and review articles. After full-text evaluation, data were independently extracted by the authors for further assessment of qualitative and quantitative evidence synthesis. The following information was extracted from each study: name of author, journal and year of publication, study type, number of patients per study, patient age, prostate volume, prostate-specific antigen (PSA), international prostate symptom score (IPSS), IPSS-quality of life (QoL), maximum urinary flow rate (*Q*_max_), postvoid residual (PVR), follow-up period, secondary interventions, and ejaculatory and sexual function. In accordance with the PRISMA criteria, Fig. 1 was included to delineate our article selection process.

## Results

A total of 15 articles were eligible for inclusion for all 3 MIS techniques. iTIND: a multicenter single-arm prospective study with 1- and 2 year follow-up [8, 9]. Urolift: a single-center retrospective study [10], a multicenter blinded randomized controlled trial (RCT) with 5 year follow-up [11–13], a multicenter non-blinded RCT with 2 year follow-up [14, 15], a retrospective study with prospectively collected data [16], and a multicenter non-randomized prospective study [17]. Rezūm: a multicenter blinded RCT with 4 year follow-up [18], a cross-over study from the RCT [19], a multicenter retrospective study [20], a single-center retrospective study [21], and a prospective nonrandomized pilot study [22]. Ultimately, a total of 81, 418, and 505 patients were recruited for iTIND, Urolift and Rezūm, respectively. The study selection process is outlined in Fig. 1.

## Temporary implantable nitinol device (iTIND)

### Surgical technique

iTIND is a second-generation mechanical device that consists of three struts with double intertwined nitinol wires configured as a tulip-shaped stent. The struts are located at the 12, 5 and 7 o’clock positions which are cranially linked together to support their exertion on the urethral mucosa when expanded and to avoid potential bladder mucosal injuries. As in the first-generation device, it includes an anchoring leaflet and a distant nylon wire for removal [23]. The insertion procedure is performed through a transurethral approach with a rigid cystoscope and routinely done under intravenous sedation; however, it may also be positioned under local anesthesia. The folded device is preloaded into a 14-Fr delivery system and deployed into a full bladder. Once the surgeon perceives friction reduction against the sheath´s internal surface, the plastic sheath around the nylon wire is removed and the knot at the wire's end is severed. The cystoscope is reinserted to place the device cranial to the verumontanum and at 6 o'clock distal to the bladder neck. Through this mechanism, the device is securely positioned and held in place, while the three elongated struts release outward pressure towards the prostatic tissue and bladder neck to induce prostatic tissue necrosis, prostate reshaping and thus eliminating the prostatic obstruction.

The iTIND device is removed after 5 days of implantation by any of the two following methods. The first removal technique can be conducted under topical anesthesia in an ambulatory setting by pulling the nylon wire into a 20–22 Fr. open-ended catheter with the aid of the semi-rigid double wire or Snare. The device is pulled from the nylon wire to be withdrawn into the catheter lumen and eventually removed. The second method is performed under general anesthesia with a rigid cystoscope using the Snare. The nylon wire anchored to the device is inserted into the cystoscope sheath which is then inserted into the urethra to be closed and removed under direct visualization.

### Clinical results

Clinical evidence for the safety and efficacy of the second-generation iTIND device is mainly based on two studies [8, 9]. In a single-arm, multicenter, international prospective study, Porpiglia et al. evaluated 81 patients with BPH-related LUTS who were treated with iTIND and followed for 1 year [8]. All implantations were successful with no transoperative complications reported, patients were discharged on the same day of surgery, and the devices were retrieved at a mean 5.9 days after insertion. In comparison to baseline, none of the 61 patients who completed the 12 month follow-up reported sexual or ejaculatory dysfunction, and all complications graded as ≤ 2 Clavien-Dindo were self-limiting. In terms of functional results, significant improvements were recorded in IPSS score, QoL, *Q*_max_ and PVR from baseline to 1 year follow-up. Mean *Q*_max_ improved from 7.3 ± 2.6 to 14.9 ± 8.1 ml/s, IPSS score from 22.5 ± 5.6 to 8.78 ± 6.4, QoL from 4 (2–5) to 1 (0–4), and PVR from 77.3 ± 55.2 to 34.0 ± 54.1 ml. The treatment failure rate was 5% (4/81), two patients required TURP, one patient combined therapy with α-blocker and 5α-reductase inhibitor while one patient only α-blocker.

Two year outcomes were reported by Kadner et al., where a significant reduction in symptoms and an improvement in urinary flow were maintained: IPSS score improved to 8.5 ± 5.51, QoL to 1.76 ± 1.32, and *Q*_max_ to 16.0 ± 7.43. No deterioration in sexual or ejaculatory function was recorded, and five patients underwent surgery due to treatment failure of which four had median lobes [9]. Table 1 summarizes the studies evaluating iTIND for the treatment of LUTS associated with BPH.

**Table 1:** Summary of studies evaluating iTIND for the treatment of BPH-related LUTS

Studies	Study type	Patients	Patient age (years)	Prostate volume (cc)	PSA (mg/dl)	IPSS	IPSS-QoL	*Q*_max_ (cc/s)	PVR (mL)	Implantation duration (days)	Follow-up (months)	Ejaculatory/sexual function—secondary interventions
Baseline Characteristics		81	65 (45.5–84.5)	40.3 ± 12.3	1.7 ± 1.4	22.5 ± 5.6	4 (2–5)	7.3 ± 2.6	77.3 ± 55.2			
Porpiglia et al. BJUI 2019 [8]	Single-arm, multicenter, international prospective	67	–	–	–	8.78 ± 6.4^*^	1 (0–4) ^*^	14.9 ± 8.1^*^	34.0 ± 54.1^*^	5.9 ± 1.1	12	None of the 61 sexually active patients reported sexual or ejaculatory dysfunction Two (2.4%) patients required TURP and two (2.4%) medical therapy for BPH
Kadner et al. WJU 2020 [9]	*MT-02 study* Single-arm, multicenter, international prospective	51	–	–	–	8.51 ± 5.51^*^	1.76 ± 1.32^*^	16.0 ± 7.43^*^	14.3 ± 24.1^*^	5.7 ± 0.9	24	No deterioration in sexual and ejaculatory function 12 (14.8%) failed treatment, 5 required surgery and 4 of these had a median lobe

*IPSS* international prostate symptom score, *PSA* prostate-specific antigen, *PVR* post void residual, *QoL* quality of life, *Q*_max_ maximum urinary flow rate

*Indicates significant finding compared to baseline value

In conclusion, iTIND represents a viable option for patients seeking low-risk minimally invasive therapy, particularly in sexually active patients seeking ejaculation and sexual function preservation. Although three further prospective studies are being carried out and longer follow-up is warranted, it seems justifiable to recommend this approach in patients who desire significant symptom relief and are reluctant to accept long-term medical therapy [24].

### EAU guideline summary of evidence and recommendations

- No EAU recommendation since the technique is under investigation requiring RCTs against a reference technique. Secondary studies are needed to confirm the reproducibility and generalizability of the first pivotal study [25].

### AUA guideline statement

- Technique not included in AUA guideline.

## Prostatic urethral lift (Urolift)

### Surgical technique

The prostatic urethral lift (PUL) or Urolift approach includes permanent tissue-retracting implants which aim to create a continuous anterior channel through the prostatic urethra extending from the bladder neck to the verumontanum. It is ideally suited for patients with prostate volumes between 20 and 70 cc and typical lateral lobe obstruction. Under local anesthesia and cystoscopic visualization, implants consisting of a capsular nitinol anchor (0.6 mm in diameter and 8 mm in length) and an adjustable, non-absorbable PET monofilament are placed anterolaterally at the 2 and 10-o´clock positions to ensure neurovascular bundle and dorsal venous plexus preservation. The implants are designed to compress the obstructive tissue and therefore expand the prostatic urethra. Relative contraindications include a prominent median lobe, a high bladder neck, and prostates larger than 100 cc. Nonetheless, several studies have shown good results as well as high patient safety in cases with protruding middle lobes and severe obstruction [16, 26, 27].

### Clinical results

The safety and efficacy of the PUL procedure has been demonstrated in multiple studies [10–12, 14–17, 26]. The L.I.F.T. study is a prospective, randomized, sham controlled, blinded clinical trial performed across 19 centers in the United States, Canada and Australia with a 5 year follow-up. It demonstrated the superiority of PUL in comparison to a sham cystoscopic procedure for the improvement of LUTS and health-related quality of life. There were significant improvement in IPSS, QoL and *Q*_max_ from baseline to 3 years follow-up but not in PVR [11]. PUL efficacy remained durable through 5 years with overall IPSS, QoL and *Q*_max_ improved by 36%, 50% and 44%, respectively. Surgical retreatment for failure to cure was 13.6% with no adverse effects from reinterventions. Furthermore, there was no significant deterioration in erectile and ejaculatory function over the course of 5 years [12]. Fifty-three patients with moderate-to-severe LUTS who underwent a sham procedure in the L.I.F.T study were enrolled in a crossover study in which they received PUL treatment and were followed for 2 years [13]. The IPSS, QoL and *Q*_max_ rates improved 36%, 40% and 77% from baseline, respectively, and only four patients (8%) progressed to TURP, while one (2%) required additional PUL implants.

The prospective, randomized, controlled, non-blinded BPH6 study compared PUL to TURP at 10 European centers with regard to symptoms, recovery, sexual function, continence, safety, quality of life, sleep and overall perception [14, 15]. This non-inferiority study including 80 patients demonstrated significant improvements in IPSS, QoL, and *Q*_max_ in both arms throughout the 2 year follow-up. Although changes in IPSS and *Q*_max_ were superior in the TURP arm, QoL improvements were not statistically different and PUL resulted in superior quality of recovery, sleep, ejaculatory function and performance [15].

In a prospective and multicentric study, Sievert et al. investigated PUL outcomes for the treatment of LUTS in 86 patients who were offered PUL as an alternative procedure to TURP. Significant improvements were observed in mean IPSS (51%), QoL (52%), PVR (70%) and *Q*_max_ (27%) which were maintained over the 2 year follow-up. Eleven (12.8%) patients reported persistent LUTS of which 9 were satisfactorily retreated with TURP and 1 with new PUL implants [16].

In a prospective and nonrandomized study across 6 Australian institutions, Chin et al. treated 64 men with PUL who were followed for 2 years. The IPSS score was reduced by 42%, *Q*_max_ improved by ≥ 30%, sexual function was not compromised and erectile function was slightly increased compared with baseline [17]. Table 2 summarizes the studies evaluating PUL for the treatment of LUTS associated with BPH.

**Table 2:** Summary of studies evaluating prostatic urethral lift (Urolift) for the treatment of BPH-related LUTS

Studies	Study type	Patients	Patient age (years)	Prostate volume (cc)	PSA (mg/dl)	IPSS		QoL		Q_max_ (cc/s)		PVR (mL)		Follow-up (months)	Ejaculatory/sexual function —secondary interventions
						Baseline	Last follow-up	Baseline	Last follow-up	Baseline	Last follow-up	Baseline	Last follow-up		
Kim et al. 2019 [10]	Retrospective single center	32	67 ± 7	50 ± 7	–	19.3 ± 2.4	11.2 ± 1.7^*^	4.4 ± 0.6	1.7 ± 0.6^*^	12.1 ± 2.4	15.3 ± 1.4^*^	–	–	12	No ejaculatory or sexual dysfunction No patients retreated
Sievert et al. 2018 [16]	Multicenter retrospective from a prospectively collected database	86	66.2 ± 11.5	43 ± 18.8	–	20.8 ± 6.52	10.1 ± 3.9^*^	4.1 ± 1.22	1.9 ± 0.9^*^	11.2 ± 3.2	14.2 ± 3.2^*^	149.5 ± 251.5	44.6 ± 42.3^*^	24	Sexual and ejaculatory function was unchanged or improved Eleven (12.8%) patients retreated, 9 TURP, 1 TURP and 1 declined further treatment
Roehrborn et al. 2017 [12]	LIFT study multicenter international blinded RCT	206 total 140 Urolift 66 control	67 ± 8.5	44.6 ± 12.5	2.3 ± 1.98	22.3 ± 5.4	14.5 ± 8.3^*^	4.6 ± 1.1	2.54 ± 1.76^*^	7.9 ± 2.4	11.08 ± 4.7^*^	–	–	60	No ejaculatory or sexual dysfunction Surgical retreatment for failure was 13.6% at 5 years with 6 (4.3%) receiving additional PUL and 13 (9.3%) TURP/laser ablation
Roehrborn et al. 2015 [11]	LIFT study multicenter international blinded RCT	206 total 140 Urolift 66 control	67 ± 8.5	44.6 ± 12.5	2.3 ± 1.98	22.3 ± 5.4	12.7 ± 7.9^*^	4.6 ± 1.1	2.2 ± 1.6^*^	7.8 ± 2.4	11.8 ± 4.8^*^	85.9 ± 68.9	73.3 ± 90.1	36	No ejaculatory or sexual dysfunction 15 patients (10.7%) surgically retreated, 9 TURP and 6 PUL implant
Rukstalis et al. 2016 [13]	LIFT study crossover multicenter international blinded RCT	51	64 ± 7.8	40.5 ± 9.9	2.16 ± 1.75	25.4 ± 5.5	15.2 ± 7.2^*^	4.8 ± 1.1	2.6 ± 1.5^*^	7.9 ± 2.5	12.2 ± 5.8^*^	88.1 ± 70.4	56.7 ± 58.7	24	No ejaculatory or sexual dysfunction 10 misplaced implants encrusted and 3 removed
Gratzke et al. 2016 [15]	BPH6 study multicenter international non-blinded RCT	80 total 45 Urolift 35 TURP	63 ± 6.8	38 ± 12	2.4 ± 1.8	21.9 ± 5.7	12.2 ± 8.9^*^	4.7 ± 1.1	2.1 ± 1.6^*^	9.4 ± 3.5	14.3 ± 5.3^*^	80.5 ± 61	69.9 ± 62.5	24	Ejaculatory function superior for PUL over TURP and erectile function preserved Six (13.6%) patients required retreatment
Sonksen et al. 2015 [14]	BPH6 study multicenter international non-blinded RCT	80 total 45 Urolift 35 TURP	63 ± 6.8	38 ± 12	2.4 ± 1.8	21.9 ± 5.7	10.7 ± 8.1^*^	4.7 ± 1.1	1.9 ± 1.6^*^	9.6 ± 3.5	13.6 ± 5.5^*^	86 ± 72	93.7 ± 156.5	12	Erectile function was preserved and ejaculatory function experienced improvement PUL did not cause adverse effects that required retreatment or revision
Chin et al. 2012 [17]	Multicenter nonrandomized prospective	64	66.9 ± 7.3	51 ± 23	4.0	22.6 ± 5.4	12.6 ± 7.2^*^	4.9 ± 0.9	2.5 ± 1.8^*^	8.3 ± 2.2	10.3 ± 4.1^*^	54 ± 68	89 ± 104	24	No ejaculatory or sexual dysfunction 20% (13/64) required TURP, photoselective vaporization or repeat PUL over 2 years

IPSS international prostatic symptom score, PSA prostate-specific antigen, PUL prostatic urethral lift, PVR postvoid residual, TURP transurethral resection of the prostate

*Indicates significant finding compared to baseline value

### EAU guideline summary of evidence and recommendations

- PUL improves IPSS, *Q*_max_ and QoL; however, these improvements are inferior to TURP at 24 months (level of evidence (LE), 1b) [25].
- PUL has a low incidence of sexual side effects (LE, 1b) [25].
- Patients should be informed that long-term effects including the risk of retreatment have not been evaluated (LE, 4) [25].
- Offer PUL (Urolift) to men with LUTS interested in preserving ejaculatory function, with prostates < 70 mL and no middle lobe (Strong recommendation) [25].

### AUA guideline statements

- PUL may be offered as an option for patients with LUTS attributed to BPH provided prostate volume < 80 g and verified absence of an obstructive middle lobe (Moderate recommendation; LE Grade C) [28].
- PUL may be offered to eligible patients who desire preservation of erectile and ejaculatory function (Conditional recommendation; LE Grade C) [28].

## Water vapor thermal therapy (Rezūm)

### Surgical technique

The Rezūm system implements convective water vapor thermal energy generated via radiofrequency to cause immediate cell necrosis in the prostate [29]. A retractable needle is inserted into the targeted treatment area where steam at ~ 103 °C is applied in short 9 s bursts through an 18G needle [30]. The needle is comprised of 12 openings for steam emission which are positioned in a circular manner around the needle tip. The injection is performed at a 90° angle to the tissue and under cystoscopic control. The thermal energy is limited to the targeted prostatic capsular zone, resulting in a rapid change in tissue temperature to ~ 70 °C and irreversible cell death. The average treatment session requires 4.6 applications; however, the number of injections depends on the length of the prostatic urethra, presence of a median lobe, and the configuration and size of the prostatic gland [19].

### Clinical results

There are five studies reporting outcomes after Rezūm treatment [18–22]. Currently, the longest duration study is an ongoing double-blind RCT by McVary et al. with 4 year follow-up data [18]. A total of 197 patients were included, of whom 136 were randomly allocated to receive Rezūm therapy and 61 a sham/control cystoscopic procedure. Statistically significant improvements in IPSS (47%), QoL (43%), and *Q*_max_ (50%) were observed at 3 months and were sustained throughout 4 years. In total, six (4.4%) patients in whom a median lobe was identified but not treated required surgical intervention and seven (5.2%) patients initiated α-blockers during follow-up. The cross-over cohort outcomes were similar to that of the main trial where significant improvements were observed across subjective questionnaire scores and maximum urinary flow rates [19]. Dixon et al. performed a nonrandomized pilot study to evaluate the effectiveness of Rezūm therapy in 65 patients throughout 2 years [22]. Significant reductions in the IPSS (55.7%) and QoL (59%) were observed at last follow-up, a 44.5% improvement in *Q*_max_ was recorded and no clinically significant adverse effects were seen in sexual function.

Moreover, two retrospective studies have been conducted to assess Rezūm outcomes in men treated for LUTS attributed to BPH. Mollengarden et al. reported a single surgeon’s results of using the Rezūm procedure in 129 patients. Although statistically significant improvements were observed in IPSS (60%), *Q*_max_ (71.7%) and PVR (34.8%) at 6 months follow-up, the study was limited by variation in baseline characteristics, lack of standardized follow-up, and inadequate medication washout prior to the procedure. Nonetheless, these shortcomings were argued to more closely represent clinical practice patterns [21]. Other less frequently reported outcomes included reductions in prostate volume (17.9%) and PSA (14%) from baseline, 89.5% pharmacological management cessation, and 86% of patients would recommend others to undergo the procedure. Three (2.3%) patients underwent additional BPH surgery for persistent LUTS, two repeat Rezūm sessions and one photovaporization of the prostate. However, the low retreatment rates reported represent a shorter follow-up period compared to other studies and long-term data is required.

Darson et al. performed another retrospective study analyzing Rezūm outcomes in 131 patients treated in two large group-community practices. Although there was great variation in patient demographic data, no strict inclusion criteria and 12% of patients had prior surgical/MIS prostate interventions, it replicated the outcomes observed by McVary et al. in the RCT and in other studies [18]. At 12 month follow-up, the mean IPSS reduction was 45.2%, mean *Q*_max_ improved by 51.4%, mean PVR was reduced by 34.9%, and no adverse events related to sexual function were reported [20]. Table 3 summarizes the studies evaluating the effectiveness of Rezūm for the treatment of LUTS associated with BPH.

**Table 3:** Summary of studies evaluating water vapor thermal therapy (Rezūm) for the treatment of BPH-related LUTS

Studies	Study type	Patients	Patient age (years)	Median lobes treated *n*, (%)	Prostate volume (cc)	PSA (mg/dl)	IPSS		QoL		Q_max_ (cc/s)		PVR (mL)		Follow-up (months)	Ejaculatory/sexual function —secondary interventions
							Baseline	Last follow-up	Baseline	Last follow-up	Baseline	Last follow-up	Baseline	Last follow-up		
Dixon et al. 2016 [22]	Prospective nonrandomized pilot study	65	66.6 ± 7.7	14 (21.5%)	48.6 ± 20.5	3.9 ± 4.2	21.6 ± 5.5	9.6 ± 6.5^*^	4.3 ± 1.1	1.8 ± 1.4^*^	7.9 ± 3.2	12 ± 6.2^*^	92.4 ± 77.3	62.8 ± 83.9	24	No significant changes in sexual function were observed over 2 years One patient underwent TURP due to persistent LUTS
Roehrborn et al. 2017 [19]	Cross-over study from a multicenter international blinded RCT	45	63.8 ± 7.3	58 (30.8%)	44.7 ± 12.4	1.9 ± 1.4	20 ± 6.6	10.2 ± 6.2^*^	3.9 ± 1.4	2.1 ± 1.4^*^	10.1 ± 3.7	14 ± 6.4^*^	93.9 ± 77.2	84.6 ± 92	12	No de novo erectile dysfunction was reported Eight (3.7%) patients were retreated: 1 open prostatectomy, 3 repeat Rezum and 4 TURP
Darson et al. 2017 [20]	Multicenter retrospective	131	70.9 ± 9.4	54 (41%)	45.1 ± 23.4	3.5 ± 5.6	19.5 ± 6.6	10.1 ± 7.2^*^	4.3 ± 1.2	2.5 ± 1.4^*^	8.6 ± 4.9	10 ± 5	216.8 ± 286.6	77.3 ± 122.1^*^	12	No adverse sexual function events were reported Three (2%) patients underwent TURP and 1 patient had a second Rezum procedure
Mollengarden et al. 2017 [21]	Single-center retrospective	129	67.4 ± 8	85 (65.9%)	52.6 ± 17	2.45 ± 1.91	18.3 ± 7.5	6.9 ± 5^*^	–	–	10.5 ± 4.3	16.8 ± 6.9^*^	106 ± 127	73.1 ± 91.0^*^	6	Four (3.1%) patients reported erectile dysfunction and 4 anejaculation Three (2.3%) patients underwent secondary BPH surgery- two repeat Rezum and one photovaporization
McVary et al. 2019 [18]	Multicenter international blinded RCT	197 total 135 Rezum 61 Sham/control procedure	63 ± 7.1	58 (30.9%)	45.8 ± 13	2.1 ± 1.5	22.0 ± 4.8	11.4 ± 7.4^*^	4.4 ± 1.1	2.3 ± 1.5^*^	9.9 ± 2.2	13.7 ± 5.7^*^	82.4 ± 51.5	75.2 ± 69.7	48	Sexual and ejaculatory function remained unchanged over 2 years. Ejaculatory bother score improved relative to baseline over 3 years Retreatment in 6 (4.4%) patients due to median lobe and 7 (5.2%) initiated α blockers within 4 years

*IPSS* international prostatic symptom score, *PSA* prostate-specific antigen, *PUL* prostatic urethral lift, *PVR* postvoid residual, *TURP* transurethral resection of the prostate

*Indicates significant finding compared to baseline value

### EAU guideline summary of evidence and recommendations

- No EAU recommendation since the technique is under investigation requiring RCTs against a reference technique to confirm the first promising clinical results and to evaluate mid- and long-term efficacy and safety [25].

### AUA guideline statements

- Rezūm may be offered to patients with LUTS attributed to BPH provided prostate volume < 80 g (Moderate recommendation; LE Grade C) [28].
- Rezūm may be offered to eligible patients who desire preservation of erectile and ejaculatory function (Conditional recommendation; LE Grade C) [28].

## Discussion

There is significant interest in the development of minimally invasive procedural treatments for LUTS due to BPH that can be performed in an office or ambulatory setting under local anesthesia, ensure rapid and durable symptom relief, and provide a favorable safety profile as an alternative to traditional TURP. Innovative intraprostatic implantable devices and tissue ablation techniques such as iTIND, Urolift and Rezūm, respectively, have gained extensive popularity, prompted a great deal of research, and presented substantial improvements in LUTS and patient satisfaction. Nevertheless, their benefits must be weighed alongside their potential limitations.

The iTIND, PUL and Rezūm approaches succeed in providing a truly minimally invasive, ambulatory patient experience with mild–moderate transient procedural complications. The most common perioperative adverse effects included self-limiting hematuria, dysuria, urgency, pelvic pain and urinary tract infection which mainly occurred in the short-term and were satisfactorily resolved within 3 weeks of treatment [8, 11, 12, 18]. In terms of functional outcomes, significant improvements in IPSS, QoL, *Q*_max_ and PVR were recorded within 3 months of treatment which were maintained throughout follow-up [8, 9, 11, 12, 18]. Of all the studies that recorded baseline PVR, five PUL and three Rezūm studies did not report significant and durable reductions in PVR [11, 13–15, 17–19, 22].

Sexual and ejaculatory functions remained unchanged in the vast majority of studies, with only one study reporting de novo erectile and ejaculatory dysfunction in four patients, respectively [21]. Contrarily, McVary et al. found that the ejaculatory bother score significantly improved relative to baseline over 3 years with Rezūm [18] and Sievert et al. observed that of the 11 patients reporting ejaculatory dysfunction at baseline, 3 (27.3%) patients reported improved ejaculatory function after PUL [16].

As opposed to PUL studies which included median lobe presence as an exclusion criterion, patients who underwent Rezūm therapy were not excluded and treated at the discretion of the physician. Dixon et al. showed that functional outcomes in these patients were similar to those without the presence of a median lobe and at 1 year comparable to those reported in the RCT [22]. McVary et al. found that patients with treated median lobe enlargement had objective and subjective improvements similar to those without an identified median lobe [18]. Other studies present similar findings in which functional outcome improvement is independent of prostate size and presence of median lobe [21, 31]. Notwithstanding, Darson et al. noted a mean IPSS decrease of 10.1 and 9.4 points at 3–6 months and 12 months, respectively, among 54 patients that had a median lobe [20]. In the iTIND MT-02 study, ten patients with median lobes were recruited as protocol deviators and at 1 year follow-up seven of these patients experienced reductions in IPSS and QoL of 12.3 ± 10.9 and 2.0 ± 2.1, respectively, and a mean increase in *Q*_max_ of 11.1 ± 21.8 ml/s. However, six of the seven patients failed treatment between 12 and 24 months and median lobe presence was found to be a statistically significant predictor for treatment failure [9].

Most studies limited their prostate sizes to 80 cc with mean prostate volumes treated by iTIND, Urolift and Rezūm being 40.3 ± 12.3, 43.9 ± 15.7, and 47.4 ± 17.8, respectively. The small and medium-sized prostate glands examined in the studies and also the cohorts do not necessarily reflect the patient population being referred to TURP, endoscopic enucleation of the prostate or open prostatectomy. Therefore, further investigations are required in large-sized prostates to determine its correlation with symptom relief over time and whether it is a predictive factor for treatment response.

Retreatment rates in the PUL studies ranged from 10 to 20% with only one study reporting no retreated patients [10], while after Rezūm therapy these were < 5% [18–22]. Nevertheless, these rates are acceptable provided patients can be satisfactorily retreated with minimally invasive approaches and are not initially exposed to more invasive treatments such as TURP or open prostatectomy.

## Conclusion

The successful outcomes observed in the iTIND, PUL and Rezūm studies for the treatment of LUTS resulting from BPH is a stepping stone towards the further adoption of such minimally invasive procedures aiming to guarantee a short recovery time and return to normal activity while also maintaining sexual and ejaculatory functions intact. However, longer follow-up and the results of ongoing clinical trials are required to verify whether their advantages are sufficient to convince practitioners, patients and insurers to ensure their long-term usage and applicability in daily clinical practice.
